# Endourology and Benign Prostatic Hyperplasia in COVID-19 Pandemic

**DOI:** 10.1590/S1677-5538.IBJU.2020.S104

**Published:** 2020-07-27

**Authors:** Alexis M. Alva Pinto, Mariano Sebastián González

**Affiliations:** 1 Clínica Delgado Department of Urology Lima Peru Department of Urology, Clínica Delgado, Lima, Peru; 2 Hospital Italiano Department of Urology Buenos Aires Argentina Department of Urology, Hospital Italiano, Buenos Aires, Argentina

**Keywords:** Prostatic Hyperplasia, COVID-19 [Supplementary Concept], Pandemics

## Abstract

The new disease COVID-19 pandemic has completely modified our lifestyle, changing our personal habits and daily activities and strongly our professional activity.

Following World Health Organization (WHO) and health care authorities around the World recommendations, all elective surgeries from benign diagnose procedures must be postponed and imperatively continue working on emergent and oncological urgent pathologies. Surgical elective treatment of benign prostatic hyperplasia (BPH) is not considered as a priority.

During BPH endoscopic surgeries, urine and blood are mixed with the irrigation liquid implying a risk of viral presence. Furthermore, a steam and smoke bubble is being accumulated inside the bladder implying the risk of splashing and aerosols. The risks of other viral infections have been identified during endourological procedures and they are related to splashing events. Several studies observed 33-100% of splashing on goggles.

All BPH endoscopic procedures must be postponed. In case of complete urinary obstruction, this event can be adequately treated by urethral or suprapubic catheter under local anesthesia.

As soon as local COVID-19 prevalence decreases, endourological procedures could be restarted. As protocols are being validating around the World to redeem elective surgeries, a symptomatic obstructed patient could be operated knowing his COVID-19 status with a molecular PCR, a cleaned epidemiological interview with a normal preoperative protocol.

If patient is COVID-19+, surgery must be delayed until complete recovery, because mortality could increase as Lei from Wuhan describes.

Informed consent must include risks of complications related to COVID-19 disease. Surgery must be performed by an experienced surgeon in order to avoid increase of operating time and risks of complications.

Surgical approach of BPH must be considered depending on availability of disposable material, infrastructure, and the epidemiological COVID-19 status of your area. The main aim is patients and healthcare staff safety.

## INTRODUCTION

The coronavirus named SARS-CoV-2 is causing an outbreak of a new disease COVID-19. This pandemic has completely modified our lifestyle, changing our personal habits and daily activities and strongly our professional activity. At this point, current medical data and its clinical applications as suggested by conventional guidelines has had to be adapted to the feasibility of this new state of affairs.

Nowadays, we face a new disease on Earth and there are not high level of evidence recommendations to deal with. Furthermore, during this pandemic, we have to follow expert recommendations modifying our urological indications and out-patient and in-patient schedules prioritizing emergent and urgent procedures in order to avoid a life-threatening situations or disease progression and/or maintain patient and sanitary staff safety with novels biosecurity protocols.

Following World Health Organization (WHO) and health care authorities around the World recommendations, all elective surgeries from benign diagnose procedures must be postponed and imperatively continue working on emergent and oncological urgent pathologies. Unfortunately, in this context, surgical elective treatment of benign prostatic hyperplasia (BPH) is not considered as a priority.

However, this is a dynamic situation and information is evolving rapidly, so it is important to consider the status of your institution and locality to continue or recover your usual practice as proposed by the American College of Surgeons ( 1 ).

### Risks during endourological procedures:

SARS-CoV-2 is a RNA virus belonging to the beta coronavirus family able to infect the upper respiratory tract. Spread mechanisms have been studied and identified from human-to-human respiratory interactions to airborne and fomites transmissions ( 2 – 8 ).

In a hospital setting, knowing the extent of environmental contamination of SARS-CoV-2 in hospital wards (HW), operating room (OR) and intensive care units (ICU) is critical for improving safety practices for sanitary staff. Airborne and touching-fomites transmission have been observed in COVID dedicated ICUs and HWs ( 6 ).

Some specific aerosol-producing procedures as endotracheal intubation, bronchoscopy, open suctioning, administration of nebulized treatment, manual ventilation before intubation, turning the patient to the prone position, disconnecting the patient from the ventilator, non-invasive positive-pressure ventilation, tracheostomy, and cardiopulmonary resuscitation, are considered high-risk situations ( 4 , 6 ). Furthermore, all aerosolization procedures are a security key point to be considered.

Carcinogenetic hazard and risk of transmission of several bacteria and virus as *corynebacterium* , tuberculosis, human immunodeficiency virus (HIV), human papillomavirus (HPV) and Hepatitis B have been described in different studies related to surgical smoke ( 9 – 13 ). At that point, many questions have been posed about bio-security in open, endoscopic and laparoscopic / robot assisted surgeries ( 13 – 15 ).

Some risks during endourological procedures have been identified and they are related to splashing events and smoke/aerosolization producing procedures. SARS-CoV-2 RNA has been found also in blood, feces and urine implying a potential risk of transmission during endoscopic surgeries ( 16 – 21 ).

All surgeons are aware of risks of viral transmission in blood splashing, however they are underestimated. Those who use routinely goggles notice these droplets over them. Several studies observed 33-100% of splashing on goggles ( 22 – 24 ) as shown in Table-1 . Moreover, not only on the chief surgeons but in assistants too, so eye protection is strongly suggested in each endourological procedure ( 24 ).

**Table 1:** Splashing blood events on eye protection.

Author	Procedure	What was examined	Splash event
Davies and Harrison ( 22 )	Transuretral Resection	Blood on Spectacles	100%
Muir and Davies ( 23 )	Video Resection	Blood droplets on goggles	66%
Wines et al. ( 24 )	Video TUR	Blood droplets on goggles	33%
Wines et al. ( 24 )	Flexible URS	Blood droplets on eyes shield	58%

In relation to aerosolization and smoke produced, the hazard in endoscopic procedures are moderate compared with the biological and carcinogenetic risks observed in open and laparoscopic surgeries ( 13 ). Laparoscopic procedures are related with high concentrations of surgical smoke and 10 times aerosol potential exposure ( 15 ) but at the same time they are a natural physical barrier, this aerosolization hazard has the opportunity of being very well controlled. Current ablative BPH endoscopic technologies deliver thermal energy to cut, resect, vaporize and coagulate prostatic tissue. These well-known technologies use electrical (monopolar and bipolar) and laser (holmium, thulium, KTP, diode) sources of energy allowing a huge variety of procedures including transurethral resection, plasma bipolar and laser vaporization, and laser enucleation. During these surgeries, urine and blood are mixed with the irrigation liquid implying a risk of viral presence. Furthermore, a steam and smoke bubble are being accumulated inside the bladder implying the risk of splashing and aerosols even if no COVID-19 transmission has not been reported by this way.

### COVID-19 and Benign Prostatic Hyperplasia (BPH)

Since hospitals will face a huge demand of resources to fight against a possible surge of COVID-19 cases, elective surgeries of benign pathology have been recommended to be delayed until the strain on the hospital system from COVID-19 decreases. Following this rules, all BPH procedures as TURP, HoLEP, ThuLEP, PVP, etc. must be postponed. In case of complete urinary obstruction, this event can be adequately treated by urethral or suprapubic catheter under local anesthesia ( 25 , 26 ).

As soon as local COVID-19 prevalence decreases ( 26 ), endourological procedures could be restarted. As protocols are being validating around the World to redeem elective surgeries, a symptomatic obstructed patient could be operated knowing his COVID-19 status with a molecular PCR, a cleaned epidemiological interview with a normal preoperative protocol. If patient is COVID-19+, surgery must be delayed until complete recovery. Informed consent must include risks of complications related to COVID-19 disease. Ti et al. reported up to 20% mortality rate in asymptomatic COVID-19+ patients which were unintentionally programed for elective surgery ( 27 ).

Including non COVID-19, all patients must be considered as suspected ones. At this point, surgical staff including scrub nurse and anesthesiologist must wear a level 2 or 3 PPE. Initially, only the anesthesiologist and the assistant must be inside the OR with the patient. Once the patient is anesthetized, surgical staff can enter into the OR. It is important to minimize the number of strictly needed people and to count with all required materials in order to avoid frequent door openings ( 28 ). Surgery must be performed by an experienced surgeon in order to avoid increase of operating time and risks of complications. During surgery, in order to minimize smoke or aerosol risks, surgeon must be attentive to exchanges of equipment, to systematically aspirate the gas bubble and the outflow drainage connected to the central aspiration system.

## CONCLUSIONS

Endoscopic procedures apparently are not as hazardous as open or laparoscopic approaches because aerosolization and smoke carcinogenetic risks and viral transmission are less frequent.

Surgical approach of BPH must be considered depending on availability of disposable material, infrastructure, and the epidemiological COVID-19 status of your area. The main aim is patients and healthcare staff safety.
